# Motivation of UK graduate students in education: self-compassion moderates pathway from extrinsic motivation to intrinsic motivation

**DOI:** 10.1007/s12144-021-02301-6

**Published:** 2021-09-22

**Authors:** Yasuhiro Kotera, Elaina Taylor, Dean Fido, Dan Williams, Freya Tsuda-McCaie

**Affiliations:** 1 College of Health, Psychology and Social Care, University of Derby, Derby, UK; 2 College of Arts, Humanities and Education, University of Derby, Derby, UK

**Keywords:** Academic motivation, Intrinsic motivation, Academic, Engagement, Self-compassion, Self-criticism

## Abstract

Academic motivation is recognised as a key factor for academic success and wellbeing. Highly motivated students actively engage with academic activities and maintain good wellbeing. Despite the importance of motivation in education, its relationship with engagement and wellbeing remains to be evaluated. Accordingly, this study explored the relationships between motivation, engagement, self-criticism and self-compassion among UK education postgraduate students. Of 120 postgraduate students approached, 109 completed three self-report scales regarding those constructs. Correlation, regression and moderation analyses were performed. Intrinsic and extrinsic motivation were positively associated with engagement, whereas amotivation was negatively associated with it. Engagement positively predicted intrinsic motivation. Self-criticism and self-compassion moderated the pathway from extrinsic motivation to intrinsic motivation: higher self-criticism weakened the pathway, while higher self-compassion strengthened it. Findings suggest the importance of engagement in relation to cultivating intrinsic motivation of education students. Moreover, enhancing self-compassion and reducing self-criticism can help transfer extrinsic to intrinsic motivation.

## Introduction

Poor mental health is well-documented in higher education students (Castillo & Schwartz, 2013; Conley et al., 2015; Hunt & Eisenberg, 2010; Sharp & Theiler, 2018). Students experience high levels of anxiety, depression, and stress across a wide range of disciplines and levels of studies (Larcombe et al., 2016), which leads to diverse negative outcomes such as lower achievement and retention (Dekker et al., 2020; Eisenberg et al., 2009). This trend has become intensified more recently. The mental health of students has been highlighted as a pressing concern due to the effects of the coronavirus disease 2019 (COVID-19) and the associated impact this has had on the attainment and educational requirements of students (Sahu, 2020). Poor mental health in students needs to be addressed urgently.

Research indicates that students’ mental health is associated with academic engagement (Liébana-Presa et al., 2014; Rogers et al., 2017; Suárez-Colorado et al., 2019; Turner et al., 2017) that refers to the extent to which a student is driven to make an effort in their studies (Newman & Schwager, 1992). Students with higher scores on measures of mental wellbeing tended to have higher levels of academic engagement (Datu & King, 2018; King et al., 2015). When examining 338 Philippine university students, mental wellbeing longitudinally predicted higher engagement and lower disengagement, indicating potential for developing future interventions aimed at improving engagement (King et al., 2015). Although several studies have examined the relationship between mental health and engagement internationally, less research has been conducted with UK students, indicating a need for evaluation.

Students with greater wellbeing also evidence greater academic motivation (Datu, 2018; Isen & Reeve, 2005; Kotera, Green, & Sheffield, 2019a; Lewis et al., 2009). Datu (2018) outlined that low-arousal positive emotions such as feelings of harmony and peacefulness were related to more helpful, autonomous types of learning. Academic motivation (a cognitive and behavioural drive to meet academic goals; Kovach, 2018) is associated with a range of academic measures of success, and can be understood within the context of Self-Determination Theory (SDT).

### Overview of Student Motivation Conceptualised by Self-Determination Theory

The SDT conceptualises motivation as intrinsic (motivated by satisfaction or pleasure), extrinsic (motivated by external or instrumental factors) or amotivation (lack of motivation) (Deci & Ryan, 1985; Ryan & Deci, 2017). These concepts are based on a self-determination continuum, wherein intrinsic motivation relates to more autonomous, volitional forms of motivation. Intrinsically motivated individuals complete tasks based on the satisfaction of the task itself, without additional reward or associated consequence (Kotera et al., 2021a, b; Ryan & Deci, 2000). Intrinsically motivated students find academic activities meaningful, and actively seek learning opportunities beyond mandatory learning and assessments. Conversely, extrinsically motivated individuals are prompted to complete tasks based on external factors, and satisfaction comes from extrinsic consequences (i.e., receiving rewards or to avoid punishment), rather than the task itself (Deci & Ryan, 2002). Therefore, extrinsically motivated students may be prompted to complete tasks based on upcoming deadlines or to avoid poor grades. It is equally important to note that intrinsic motivation and extrinsic motivation are not in an either-or relationship: one student can have both types of motivation high or low (Deci & Flaste, 1996; Derfler-Rozin & Pitesa, 2021).

Deci and Ryan (2002) also outlined a third motivational concept, in which individuals are neither extrinsically nor intrinsically motivated—amotivated individuals. People with this type of motivation cannot perceive the link between their own behaviour and behavioural outcomes (Deci & Ryan, 1985; Ryan & Deci, 2017), and experience feelings of lack of control likened to learned helplessness (Legault et al., 2006). Amotivated students may have little interest, place low value on learning activities and show minimal engagement.

### Relationship between Motivational Styles and Student-Related Outcomes

In students, these three forms of motivation are differentially associated with academic success. Students who are intrinsically motivated flourish across academic settings (Goldman et al., 2017). Intrinsic motivation is associated with better academic performance (Lepper et al., 2005), higher retention rates (Vallerand, 1997) and greater wellbeing (Ryan & Deci, 2000; ten Cate et al., 2011). Conversely, extrinsic motivation is associated with poorer academic outcomes such as lower learning effectiveness (Lepper et al., 2005; Zaccone & Pedrini, 2019). Similarly amotivation also leads to poorer education-related outcomes such as lower productivity and wellbeing (Legault et al., 2006).

Previously, extrinsic motivation was seen to directly contrast with intrinsic motivation, but more recent research indicates that both forms of motivation should be seen as complementary. Organismic Integration Theory (OIT), a sub-theory of SDT, explains that extrinsic motivation can support the development of intrinsic motivation (Gopalan et al., 2017; Ryan & Deci, 2017). For example, individuals who are extrinsically motivated to attend mandatory classes, may develop greater interest and intrinsic motivation as learning progresses. Helping extrinsically motivated students to develop intrinsic motivation, has been identified as an important area for educational research, highlighting the complementary nature of motivation (Ommering & Dekker, 2017).

These findings facilitated researchers and educators to develop means to augment students’ intrinsic motivation. Much research on fostering intrinsic motivation in students has focussed on factors external to the student, such as learning environment or the role of teachers (Bolkan & Goodboy, 2015; Guay et al., 2008; Niemiec & Ryan, 2009; Orsini et al., 2015; Serin, 2018). In comparison, less research has examined students’ internal factors or traits, such as self-compassion, which may encourage intrinsic motivation through greater internalisation of tasks, and better self-regulation. A flexible, self-regulated approach to learning is crucial (Neely et al., 2009) particularly for long-term goals, such as completion of an undergraduate degree (Karlen et al., 2019). This may include understanding when to pursue challenging tasks, or when to engage in more self-compassionate learning strategies.

### Self-Compassion and Motivation in Students

Self-compassion is defined as self-acceptance, achieved through 1) treating oneself kindly during times of perceived self-inadequacy, 2) recognition of common humanity and a connection with others despite perceived isolation, and 3) managing negative inward-facing thoughts such as self-pity (Neff, 2003).

Previous research has indicated that self-compassion is associated with greater motivation to learn (Neff et al., 2005). For instance, students with higher self-compassion more flexibly engage in working towards goals (Neely et al., 2009), had less negative reactions to feedback (Adams & Leary 2007; Neff et al., 2005) and show greater mastery orientation (motivation through curiosity and self-development) compared to individuals with lower self-compassion (Neff et al., 2005). More specifically, individuals with greater self-compassion are more likely to be intrinsically motivated (Kotera & Ting, 2019). This may be because self-compassionate individuals are less likely to be affected by external markers of success (engaging less in external performance evaluations) and instead, focus on maximising their potential, wellbeing, and being kind to the self in instances of failure (Kotera, 2021; Neff et al., 2005).

Interventional studies have indicated that self-compassion can be fostered in students (Dundas et al., 2017; Neff et al., 2007; Shapiro et al., 2005) to improve motivational outcomes: A two-week clinical psychologist-led intervention, consisting of three 90-min sessions and daily audio-guides, significantly increased self-compassion in university students, with sustained high levels of self-compassion at six-month follow-up (Dundas et al., 2017). Increases in self-compassion were associated with greater motivation to learn, greater personal growth self-efficacy and healthy impulse control (Dundas et al., 2017). This study indicates the potential for intrinsic motivation to be cultivated through self-compassion interventions. However further research examining the relationship of extrinsic and intrinsic motivation to self-compassion must be explored to provide insight into the development of educational interventions promoting engagement and motivational outcomes.

### Self-Compassion and Wellbeing in Students

Additionally, self-compassion is important to examine in student populations because it is consistently associated with student wellbeing (Dundas et al., 2017; Kotera et al., 2020a, b; Kotera, Conway, & Van Gordon, 2019; Leary et al., 2007; Neely et al., 2009). Students with greater self-compassion may be able to more effectively manage negative emotions (e.g., in the face of academic adversity) and engage in self-soothing by deactivating neurological ‘defence threat systems’ (Gilbert, 2010; Kelly et al., 2009). Self-compassion was a strong positive correlate of and explained large variance in wellbeing (Neely et al., 2009). Furthermore, individuals with greater self-compassion had greater goal re-engagement (flexibility to engage in meaningful, attainable goals) (Neely et al., 2009). Exploring the relationship between self-compassion and engagement-focussing specifically on academic engagement, rather than general goal engagement, can provide beneficial findings for academic contexts.

Self-compassion can be better understood to be discussed with self-criticism; a construct commonly regarded as a counterpart of self-compassion. According to Gilbert (2014) self-criticism may activate the ‘threat system’. Self-criticism consists of two forms; 1) inadequate-self, which examines individuals’ perceptions of personal inadequacy (such as being disappointed with themselves) and 2) hated-self, examining the desire to hurt or punish the self (such as stopping engaging in self-care). Previous studies have found that both components of self-criticism are associated with depression, anxiety and stress in social work, occupational therapy and psychotherapy students (Kotera, Green, & Sheffield, 2019a, 2019b): a highly relevant construct to student mental health.

### Examining Self-Determination Theory in Education Students

The SDT has been supported in a range of educational contexts (Deci & Ryan, 2008) including school-level (Owen et al., 2014; Taylor et al., 2014), further education (Goldman et al., 2017), and higher education (Beachboard et al., 2011; Jeno, 2015) students, with particular focus on healthcare students (Orsini et al., 2015) and physical education students (Standage et al., 2005; Vasconcellos et al., 2020).

Comparably, less research has focussed on education students. Examining motivational style in education students is important as research may inform how best to support their learning to improve performance, retention and wellbeing throughout their teacher training and future teaching careers. For example, intrinsic motivation in teachers has been associated with a greater sense of wellbeing and job satisfaction (Pelletier et al., 2002). Supporting more helpful motivational patterns is particularly important due to high levels of stress in student teachers (Caires et al., 2012; Gardner, 2010; Gustems-Carnicer et al., 2019; Leung et al., 2000; Umbach & Wawrzynski, 2005), and high early-career teacher attrition (Borman & Dowling, 2008; Hwang et al., 2017). Improving retention in teaching is vital, as attrition is partially responsible for a critical shortage of teachers in certain subject areas (O’Doherty & Harford, 2018). Additionally, motivational style of teachers impacts student-related outcomes (Hein, 2012; Reeve, 2009). For instance, intrinsically motivated teachers fostered greater interest in learning in their students and endorsed adaptive classroom management styles, compared to extrinsically motivated teachers (Reeve et al., 1999; Berger & Girardet, 2020).

### Summary

Taken together, exploring factors predicting intrinsic and extrinsic motivation may help guide the development of interventions in universities aimed at improving positive outcomes such as student wellbeing, achievement, and retention. Few studies have clarified the relationship between engagement and motivation in UK students. Furthermore, modifiable factors such as self-compassion and its relationship to intrinsic and extrinsic motivation should also be examined: how self-compassion and self-criticism may strengthen or weaken the pathway from extrinsic to intrinsic motivation, illustrated by OIT, can inform educational interventions. Finally, previous research has examined the importance of intrinsic motivation in academic settings, however less research specifically relates to education/teacher training students whose motivational style may not only impact their own learning, but the future learning of their pupils.

### Aims

Therefore, this study aimed to explore relationships among motivation, engagement, self-criticism, and self-compassion in UK education students. Three research questions were considered:

- *RQ1. How is each type of motivation associated with engagement, self-criticism and self-compassion?*
- *RQ2. How is each type of motivation predicted by engagement?*
- *RQ3. Does self-criticism/compassion moderate the relationship between extrinsic motivation and intrinsic motivation?*

## Methods

### Participants

All participants were 18 years of age or older, and studying in an education programme at a UK university at the time of the study; students taking an authorised break from study were excluded on the basis that their current experiences were likely to be different from their colleagues. Participants were recruited via convenience sampling using a paper-based anonymous survey distributed by programme tutors (instead of the researchers) as a means of avoiding response biases. Of the 120 part-time graduate students who were introduced to the study, 109 (91%; 70 females, 39 males; Age 27.39 ± 7.94 years old, range 21–55 years old; 104 British, 2 other Europeans and 1 Asian) completed three scales regarding motivation, engagement and self-criticism/−compassion (see “Instrument” section below), satisfying the required sample size calculated by power analysis (84: two tails, *p* H1 = 0.30, α = 0.05, Power = 0.80, *p* H0 = 0; (Faul et al., 2009)). Additionally, demographic questions were asked: age, gender, nationality, teaching age, and weekly self-study hours. Thirty-eight students (35%) were trained to teach younger students (11–15 years old), and 71 students (65%) were trained to teach older students (16 years and older). On average, they studied 9.90 h (SD = 6.98 h) outside of the classroom per week. For one student who reported visual impairments, coloured paper was provided to aid their participation. Paper data were digitised by a research assistant, who we thank for their support with this research study. Compared with the general population of UK education students (78%; Higher Education Statistics Agency, 2018), our sample recruited slightly less females (64%). No compensation was awarded for completing the survey. In line with the study ethics (obtained from the University Research Ethics Committee prior to data collection), explanations for the withdrawal of 11 participants were not sought.

### Instruments

Students’ academic motivation was measured using the Academic Motivation Scale (AMS). The AMS measures seven types of motivation: amotivation, three types of extrinsic motivation (external, introjected and identified regulation) and three types of intrinsic motivation (knowing, accomplishing and experiencing stimulation). AMS comprises 28 items, and each type of motivation is assessed using four items on a seven-point Likert scale (1=‘Does not correspond at all’ to 7 = ‘Corresponds exactly’). There is no interpretive threshold. All seven subscales have adequate to high reliability (α = .62–.91; Vallerand et al., 1992). For the purposes of this study, the three levels of extrinsic motivation subscales were combined as ‘extrinsic motivation’, and so were ‘intrinsic motivation’ (Vallerand et al., 1992).

Utrecht Work Engagement Scale for Students (UWES-S) was used to measure students’ engagement (Schaufeli & Bakker, 2004). Seventeen items in UWES-S are categorised into three subscales: vigour (six items; e.g., ‘I can continue studying for very long periods at a time’), dedication (five items; e.g., ‘I am proud of my studies’) and absorption (six items; e.g., ‘I am immersed in my studies.’). Vigour indicates high levels of energy to make an effort in one’s academic work; dedication refers to deep involvement in one’s academic work; and absorption means positive engrossment with high concentration in one’s academic work (Schaufeli et al., 2002). All items are responded to on a seven-point Likert scale, 0 = ‘Never’ to 6 = ‘Always (everyday)’ with no interpretive threshold. Reliability for each subscale was adequate to high (α = .63–.81) (Schaufeli & Bakker, 2004).

Lastly, self-criticism and self-compassion were appraised using the Forms of the Self-Criticising/Attacking & Self-Reassuring Scale, a 22-item scale on a five-point Likert scale, 0 = ‘Not at all like me’ to 4 = ‘Extremely like me’ (Gilbert et al., 2004). The 22 items were categorised into three subscales: ‘inadequate-self’ and ‘hated-self’ comprising self-criticism, and ‘reassured-self’ corresponding to self-compassion. Inadequate-self refers to feelings of inadequacy (e.g., ‘There is a part of me that puts me down.’), hated-self to a desire to hurt or torture the self (e.g., ‘I have a sense of disgust with myself.’), and reassured-self to compassion for the self (e.g., ‘I can still feel lovable and acceptable.’). There is no interpretive threshold. Reliability for each subscale was high: α = .90 for inadequate-self, .86 for hated-self, and .86 for reassured-self (Gilbert et al., 2004).

### Statistical Analyses

First the collected data were screened for outliers and the assumptions of parametric tests. Second, correlations between their motivation, engagement, self-criticism and self-compassion were evaluated (RQ1). Third, multiple regression analyses were performed to identify significant predictors for each type of motivation (RQ2). Finally, moderation analyses were done to assess whether self-criticism and self-compassion would moderate the pathway from extrinsic motivation to intrinsic motivation (RQ3).

## Results

Analyses were conducted using IBM SPSS version 26.0 and Process Macro (Hayes, 2013). No outliers were identified. All variables demonstrated good internal reliability (α = .76–.92; Table 1).

**Table 1:** Descriptive statistics: Motivation, engagement, self-criticism, and self-compassion in UK graduate students in education (*n* = 109)

Scale	Constructs (Range)	M	SD	α
Academic Motivation Scale	Intrinsic Motivation (4–28)	18.31	4.51	.92
	Extrinsic Motivation (4–28)	20.66	4.13	.85
	Amotivation (4–28)	6.18	3.31	.76
Utrecht Work Engagement Scale-Student	Vigour (0–6)	4.11	1.07	.82
	Dedication (0–6)	4.77	1.13	.87
	Absorption (0–6)	3.49	1.40	.88
Forms of Self-criticising/Attacking & Self-Reassuring Scale	Inadequate-Self (0–36)	20.30	8.76	.89
	Hated-Self (0–32)	4.97	4.82	.84
	Reassured-Self (0–20)	19.66	7.23	.91

### Relationships among Motivation, Engagement, and Self-Criticism/-Compassion (RQ1)

As dedication and amotivation were not normally distributed (Shapiro-Wilk’s test, *p* < .05), data were square-root-transformed to satisfy the assumption of normality (Field, 2017). Pearson’s correlation was calculated (Table 2).

**Table 2:** Correlations among motivation, engagement, self-criticism, and self-compassion in UK graduate students in education (*n* = 109)

		1	2	3	4	5	6	7	8	9	10	11	12	13	14
1	Gender (0 = M, 1 = F)	–													
2	Age	.06	–												
3	Programme Level	.18	−.01	–											
4	Age of Students	−.08	−.01	−.01	–										
5	Self-Study Time	.17	.19*	.12	−.05	–									
6	Intrinsic Motivation	.13	.18	−.02	.20*	.26**	–								
7	Extrinsic Motivation	.05	−.03	.07	−.02	.13	.44**	–							
8	Amotivation	−.07	.12	−.10	.03	.04	−.19*	.04	–						
9	Vigour	.05	.05	.09	.21*	.19*	.45**	.24*	−.38**	–					
10	Dedication	−.01	−.09	.08	.22*	.20*	.47**	.28**	−.32**	.63**	–				
11	Absorption	.15	.23*	.10	.15	.38**	.53**	.19*	−.20*	.61**	.52**	–			
12	Inadequate-Self	.03	−.02	−.12	−.001	.08	.01	.20*	.31**	−.33**	−.09	−.04	–		
13	Hated-Self	−.04	.09	−.13	.11	.07	.04	.07	.24*	−.28**	−.02	.05	.66**	–	
14	Reassured-Self	−.09	−.09	.08	−.04	−.24*	.02	.01	−.21*	.28**	.11	.01	−.66**	−.64**	–

**Note.** Age of Students (0 = 11–15 years old, 1 = 16 years old or older). * *p* < .05, ** *p* < .01

Intrinsic motivation was positively associated with the age of students, self-study time, extrinsic motivation, vigour, dedication, absorption, and negatively associated with amotivation. Extrinsic motivation was positively associated with vigour, dedication, absorption and inadequate-self. Lastly amotivation was positively associated with inadequate-self and hated-self, and negatively associated with intrinsic motivation, vigour, dedication, absorption and reassured-self. The coefficients with engagement subscales (vigour, dedication, and absorption) were the highest in intrinsic motivation of the three types of motivation.

### Predictors of Motivation (RQ2)

Multiple regression analyses were conducted to explore the relative contribution of vigour, dedication, and absorption to each type of motivation (Table 3). Gender and age were entered to adjust for their effects (step one), and then vigour, dedication, and absorption were entered (step two). Adjusted coefficient of determination (Adj. R^2^) were reported to identify the degree of variance in the population. Multicollinearity was of no concern (VIF < 10).

**Table 3:** Multiple regression: Engagement to motivation among education students (*n* = 109)

	Intrinsic Motivation			Extrinsic Motivation			Amotivation		
		95% CI			95% CI			95% CI	
	β	Lower	Upper	β	Lower	Upper	β	Lower	Upper
Step 1									
Gender (0 = M, 1 = F)	.12	−.08	.36	.06	−.14	.25	−.08	−.35	.15
Age	.17	<.001	.03	−.04	−.01	.01	.12	−.01	.02
Step 2									
Gender (0 = M, 1 = F)	.09	−.09	.28	.05	−.14	.24	−.08	−.32	.14
Age	.13	<.001	.02	−.03	−.01	.01	.13	−.01	.02
Vigour	.09	−.26	.61	.10	−.29	.61	−.31*	−1.26	−.16
Dedication	.27*	.11	.94	.20	−.10	.76	.02	−.80	.25
Absorption	.29**	.10	.65	.03	−.25	.32	.04	−.28	.41
Adj. R^2^ Δ		30%			6%			13%	

β = standardised regression coefficient. *p < .05; **p < .01.

Engagement accounted for 30% (large effect size; Cohen, 1988) of the variance in intrinsic motivation, 6% (small effect size; Cohen, 1988) in extrinsic motivation, and 13% (medium effect size; Cohen, 1988) in amotivation. Dedication (*p* = .01, β = .27) and absorption (*p* = .009, β = .29) were significant positive predictors for intrinsic motivation, and vigour (*p* = .01, β = −.31) was a significant negative predictor for amotivation. No predictor was identified for extrinsic motivation.

### Moderation of Self-Criticism/Compassion on Extrinsic Motivation-Intrinsic Motivation (RQ3)

Lastly, to appraise whether self-criticism and self-compassion would moderate the relationship between extrinsic motivation and intrinsic motivation, three sets of moderation analyses were conducted, using the Model 1 in the Process macro (Hayes, 2013). The predictor variables were centred before regression analyses, to avoid multicollinearity issues.

#### Inadequate-Self

**Fig. 1:**
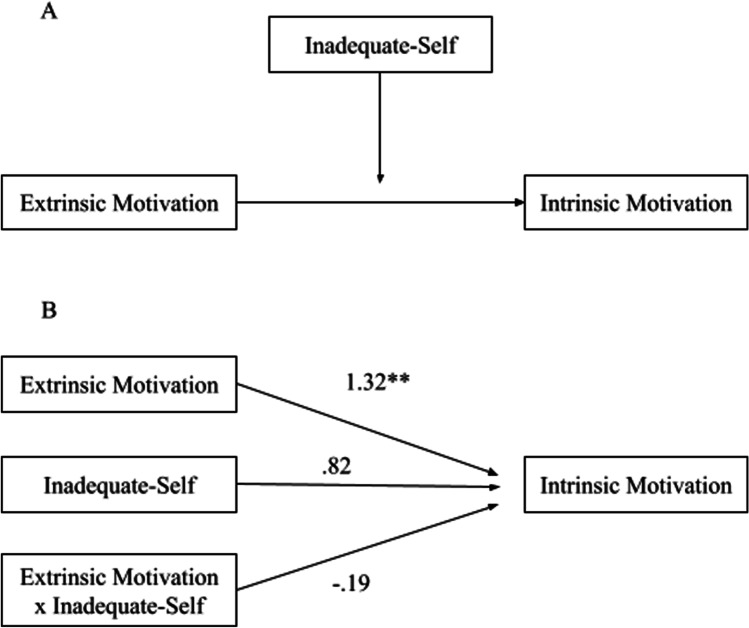
Moderation of inadequate-self on the pathway from extrinsic motivation to intrinsic motivation: conceptual diagram (panel A) and statistical diagram (panel B)

The interaction effects of extrinsic motivation and inadequate-self as predictors of intrinsic motivation were not significant (*p* = .051), indicating that inadequate-self did not moderate the pathway from extrinsic motivation to intrinsic motivation (Fig. 1).

#### Hated-Self

**Fig. 2:**
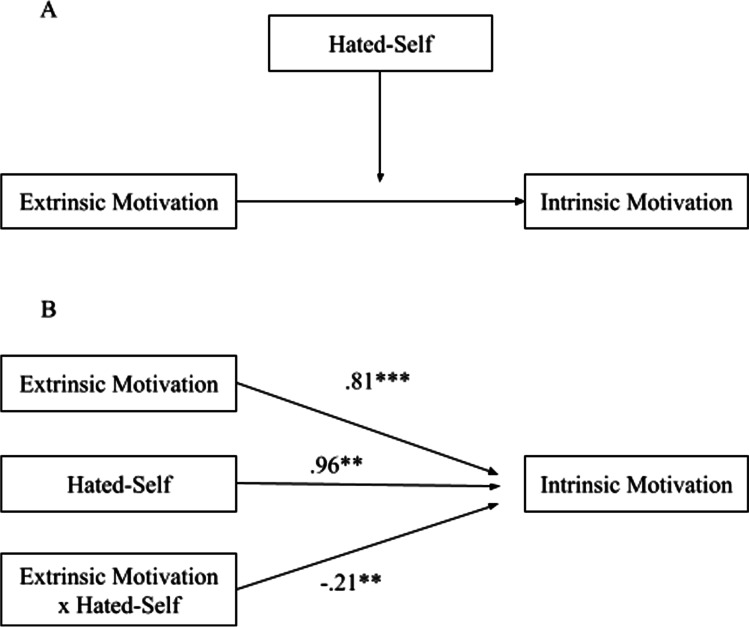
Moderation of hated-self on the pathway from extrinsic motivation to intrinsic motivation: conceptual diagram (panel A) and statistical diagram (panel B)

**Fig. 3:**
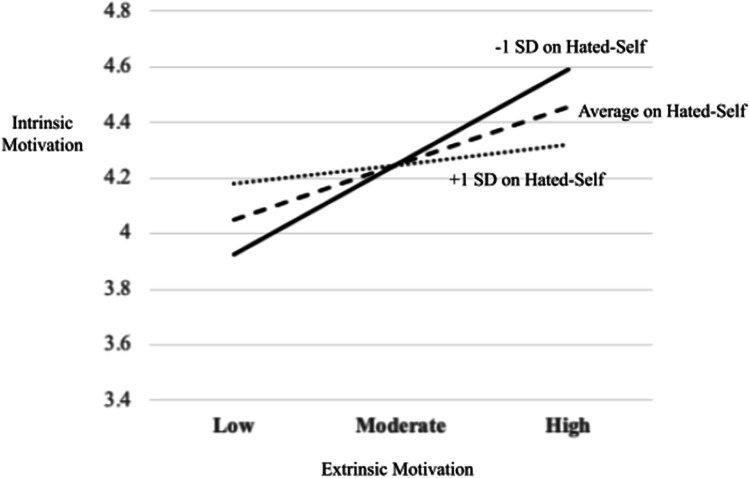
Moderating effect of hated-self on the pathway from extrinsic motivation to intrinsic motivation in education students (*n* = 109)

The interaction effects of extrinsic motivation and hated-self as predictors of intrinsic motivation were significant (*p* = .004), indicating that hated-self moderated the pathway from extrinsic motivation to intrinsic motivation (Panel B in Fig. 2). Three simple regression equations were calculated (Aiken & West, 1996) at different levels of hated-self: (i) one standard deviation below the mean hated-self score, (ii) the mean hated-self score, and (iii) one standard deviation above the mean hated-self score. The equations demonstrated a positive weakening effect of hated-self: as hated-self scores increase, the positive relationship between extrinsic motivation and intrinsic motivation becomes weaker (Fig. 3). Simple slopes analyses demonstrated that the relationship between extrinsic motivation and intrinsic motivation was significant at low and mean levels of hated-self: (i) low hated-self (*b* = .70, *t* = 5.98, *p* < .001) and (ii) mean hated-self (*b* = .43, *t* = 4.19, *p* < .001). At a high level of hated-self, it was not significant (*b* = .15, *t* = .94, *p* = .35). Johnson-Neyman significance region for hated-self was below 66.97% (73 students).

#### Reassured-Self

**Fig. 4:**
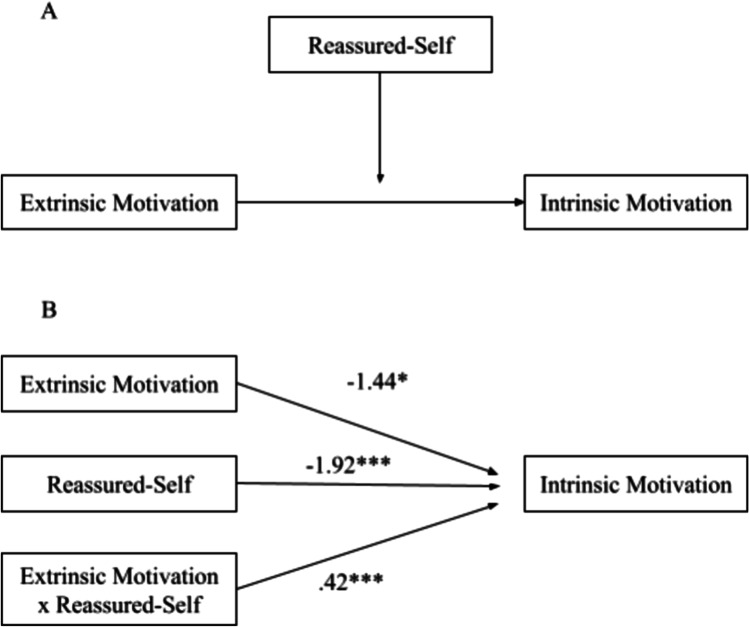
Moderation of reassured-self on the pathway from extrinsic motivation to intrinsic motivation: conceptual diagram (panel A) and statistical diagram (panel B)

**Fig. 5:**
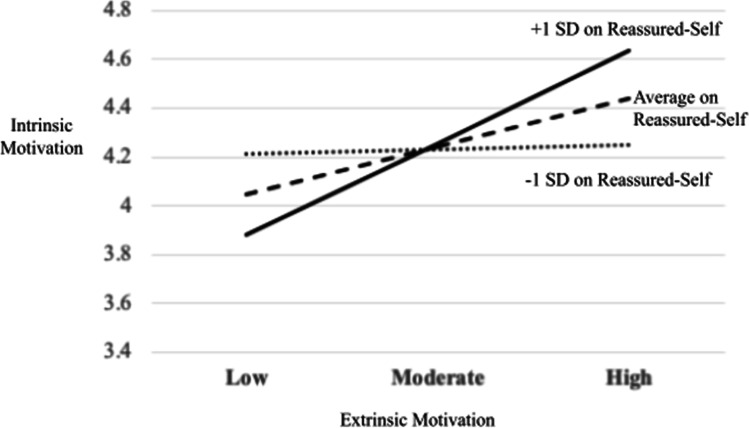
Moderating effect of reassured-self on the pathway from extrinsic motivation to intrinsic motivation in education students (*n* = 109)

The interaction effects of extrinsic motivation and reassured-self as predictors of intrinsic motivation were significant (*p* < .001), indicating that reassured-self moderated the pathway from extrinsic motivation to intrinsic motivation (Panel B in Fig. 4). Three simple regression equations (Aiken & West, 1991) demonstrated a positive enhancing effect of reassured-self: as reassured-self scores increase, the positive relationship between extrinsic motivation and intrinsic motivation becomes stronger (Fig. 5). Simple slopes analyses demonstrated that the relationship between extrinsic motivation and intrinsic motivation was significant at high and mean levels of reassured-self: (i) high reassured-self (*b* = .80, *t* = 6.38, *p* < .001) and (ii) mean reassured-self (*b* = .42, *t* = 4.23, *p* < .001). At a low level of reassured-self, it was not significant (*b* = .04, *t* = .25, *p* = .81). Johnson-Neyman significance region for reassured-self was above 74.31% (81 students).

## Discussion

This study explored relationships between motivation, engagement, self-criticism, and self-compassion in UK higher education students, and sought to delineate whether self-criticism and/or -reassurance moderated the relationship between extrinsic motivation and intrinsic motivation. After controlling for age and gender, intrinsic motivation was predicted by engagement in the form of dedication and absorption, and amotivation was predicted by engagement in the form of vigour. No manifestation of engagement significantly predicted extrinsic motivation. Moreover, the relationship between extrinsic and intrinsic motivation was moderated by hated-self and reassured-self scores, but not variation in inadequate-self. Such moderations and regression analyses are further discussed below.

This manuscript was centred around SDT, whereby motivation was conceptualised as spanning intrinsic (e.g., satisfaction, pleasure), extrinsic (e.g., external, instrumental factors), or amotivation (e.g., a lack of motivation and an inability to associate their own behaviour with behavioural outcomes) manifestations (Ryan & Deci, 2017). In line with the sub-theory, OIT (Gopalan et al., 2017; Ryan & Deci, 2017), extrinsic and intrinsic motivation are considered complementary, with extrinsic motivation thought to play a role in the development of intrinsic motivation. Given the importance of intrinsic motivation in educational settings (Deci & Ryan, 2008), developing our understanding of mechanisms by which to translate extrinsic motivation into intrinsic motivation can benefit student wellbeing and attainment. This study sought to establish whether the predictive relationship between extrinsic and intrinsic motivation could be motivated by variation in self-compassion.

### Moderation Analyses Via Self-Compassion

As predicted, there was a strong and positive relationship between self-reported extrinsic and intrinsic motivation at baseline. This relationship was moderated by variation in scores on hated-self and reassured-self, but not inadequate-self components of self-compassion. Specifically, as hated-self scores increased, the extrinsic/intrinsic motivation relationship became weaker; conversely, as the reassured-self scores increased, the extrinsic/intrinsic motivation relationship became stronger.

Self-compassion is conceptualised as self-acceptance, including allowing kind self-treatment in times of perceived self-inadequacy and managing adverse thoughts, such as self-pity (Kotera et al., 2021a, b; Neff, 2003). In educational settings, self-compassion manifests in greater motivation to learn (Neff et al., 2005), greater meaning and enthusiasm in studies (Kotera, 2018), better focus on and understanding of what is required to plan and overcome long-term challenges (Karlen et al., 2019), and the ability to learn from and navigate feedback (Leary et al., 2007; Neff et al., 2005). Thus Neely et al. (2009) argue self-compassion is vital to effective and successful education, because it facilitates a flexible and self-regulated approach to learning.

Such findings support interventional studies, which indicate the potential for self-compassion to be fostered in students to improve motivational outcomes (Dundas et al., 2017; Neff et al., 2007; Shapiro et al., 2005). For example, a targeted intervention successfully increased self-compassion in university students, which in turn was associated with greater motivation to learn and perceived personal growth (intrinsic motivation) (Dundas et al., 2017). As such, the development of educational interventions, which focus on self-compassion, may be important to promote intrinsic motivation.

### Correlates of Motivation

This research suggests that in addition to age and self-study time, intrinsic and extrinsic motivation were positively associated with vigour, dedication, and absorption, which were negatively correlated with amotivation. Of the three types of motivation, intrinsic motivation was most strongly associated with engagement. Intrinsic motivation in students has been associated with greater academic performance (e.g., Goldman et al., 2017; Lepper et al., 2005; Zaccone & Pedrini, 2019), as well as higher retention rates (Vallerand, 1997), and better student outcomes relative to extrinsically motivated students (Hein, 2012; Reeve, 2009). Increasing academic engagement via fostering intrinsic motivation also has wider implications for the benefit of student mental health and wellbeing (Datu, 2018; King et al., 2015; Liébana-Presa et al., 2014; Rogers et al., 2017; Turner et al., 2017; Suárez-Colorado et al., 2019). Thus, helping extrinsically motivated students to develop intrinsic motivation is of great importance, and enhancing students’ engagement may be effective for that purpose (Ommering & Dekker, 2017; Pelletier et al., 2002). At the same time, it is also important that educators are aware of the motivation purity bias, where extrinsic motivation is expressed, educators assume the student’s intrinsic motivation is low (Derfler-Rozin & Pitesa, 2021). Educators’ understanding of motivation is essential to gauge students’ motivation.

### Application of Results

Results of this investigation highlight the potential to increase intrinsic motivation in students, by fostering self-compassion. Fostering self-compassion may help students modify their goal orientation practices from performance to mastery orientated goals, thereby encouraging the translation of extrinsic motivation to intrinsic motivation. Performance-orientated goals are motivated by social comparison and a desire to demonstrate superiority on a task. Contrastingly, mastery-orientated goals are motivated by a desire to develop skills or understanding (Ames & Archer, 1988; Dweck, 1986). Mastery-oriented goals are associated with intrinsic motivation (Cerasoli et al., 2014; Spinath & Steinmayr 2012), and self-compassion is associated with mastery goal orientation (Babenko & Oswald 2019; Neff et al., 2005). Interventions aimed at developing self-compassion in conjunction with support for students in setting mastery-orientated goals may support intrinsic motivation.

Furthermore, fostering self-compassion may support a growth mindset, which is associated with intrinsic motivation (Dweck & Yeager 2019). A growth mindset is the understanding of intelligence as modifiable through effort rather than a fixed attribute of an individual (Dweck & Yeager 2019). Growth mindset is associated with mastery orientated goals, positive engagement with challenges, including perceived failures, and intrinsic motivation (Ng, 2018). Self-compassion, with its emphasis on self-kindness in reaction to perceived failures, may help individuals positively engage with such failures, which characterises a growth mindset. Interventions fostering self-compassion combined with psychoeducation about and teaching approaches that incorporate growth mindset may support intrinsic motivation.

Encouraging self-compassion and intrinsic motivation through feedback style may be important for postgraduate education students nurturing a growth mindset (Dweck, 2008). The development of reflective practice, including fostering self-evaluative and critically reflective teachers, is one aim of teacher training and early-career mentoring (Harrison et al., 2005). In encouraging self-reflective processes in student teachers, it may be important for mentors and educators to encourage compassionate feedback, thus preventing self-reflection from being conflated with self-criticism—which is associated with depression and poor wellbeing (Gilbert & Woodyatt, 2017). Additionally, the form of feedback provided by university teachers may be important for fostering intrinsic motivation. Providing elaborate (as opposed to simple) feedback is associated with increased motivation (Bangert-Drowns et al., 1991; Serge et al., 2013), while positive feedback strengthens motivation and learning (Henderlong & Lepper, 2002). Taken together, educators should use compassionate, elaborate and positive feedback to support students’ self-compassion and intrinsic motivation.

### Limitations

Results of this investigation are discussed in light of four core limitations, each of which should be considered when implementing observed results and conceptualising future research. First, said results are correlational and as such one cannot fully infer the effect of heightened self-compassion on the development and maintenance of intrinsic motivation. However, the results observed here map well onto both existing theory and previous peer-reviewed publications in the area, supporting their validity. Second, although the recorded sample size exceeded a-priori power analyses (n = 109), the sample, consisting of postgraduate education students, is largely heterogeneous–coming from a limited number of teaching modules at a single UK-based university. However, SDT has been reported across multiple educational contexts (Owen et al., 2014; Taylor et al., 2014; Goldman et al., 2017; Beachboard et al., 2011; Jeno, 2015), and areas of study (Orsini et al., 2015; Standage et al., 2005; Vasconcellos et al., 2020), while noting that development of intrinsic motivation may differ by culture (Liu et al., 2020). Indeed, the size of a person’s smile was associated with a level of their intrinsic motivation in cross-cultural samples (Cheng et al., 2020), however perception of facial expressions also differs cross-culturally (Jack et al., 2012). More diverse samples need to be evaluated. Third, self-report measures were used, hence the response biases might have been present (Kotera, Van Laethem, & Ohshima, 2020c). Relatedly, a ceiling effect might have been present for reassured-self (M = 19.66 of 20.00). Finally, this research was undertaken during a novel time, the COVID-19 pandemic, which has been associated with atypical variation in health and behaviour (Harper et al., 2020; Kotera et al., 2021a, b). As such, although this might represent a unique context, similar observations have been reported historically, suggesting validity in these results extending beyond this current context.

### Conclusion

Intrinsic motivation in academic settings is associated with important attainment and wellbeing outcomes, including engagement, academic success, retention, and mental wellbeing. However, it remains unknown whether modifiable factors, such as self-compassion, might help to shift one’s motivation style away from extrinsic to intrinsic motivation. Intrinsic motivation predicted engagement in the form of dedication and absorption after controlling for age and gender, and of importance, the relationship between extrinsic and intrinsic motivation was moderated by both hated-self (negatively) and reassured-self scores (positively). Results not only develop our understanding of the feasibility of developing and implementing interventions aimed at improving self-compassion in educational settings, but in doing so, suggest a potential benefit of such for increasing intrinsic motivation, which in turn might yield additional benefits in academic success and wellbeing.
